# Predictive Value of Serum IFN-γ inducible Protein-10 and IFN-γ/IL-4 Ratio for Liver Fibrosis Progression in CHB Patients

**DOI:** 10.1038/srep40404

**Published:** 2017-01-09

**Authors:** Yadong Wang, Weiyan Yu, Chuan Shen, Wei Wang, Li Zhang, Fang Liu, Hui Sun, Yajuan Zhao, Honghao Che, Caiyan Zhao

**Affiliations:** 1 Department of Infectious Disease, The Third Hospital of Hebei Medical University, Shijiazhuang, Hebei, China; 2 Division of Liver Disease, The Infectious Disease Hospital of Handan City, Handan, Hebei, China; 3 Department of Gastroenterology and Hepatology, The First Hospital of Shijiazhuang City, Shijiazhuang, Hebei, China.

## Abstract

Noninvasive serum markers for assessment of liver fibrosis in chronic hepatitis B (CHB) patients have not been well-studied. The present study was to evaluate the predictive value of serum interferon gamma-inducible protein-10 (IP-10/CXCL10) and the interferon (IFN)-γ/interleukin (IL)-4 ratio for liver fibrosis progression in CHB patients. A total of 180 CHB patients were categorized into four groups: no fibrosis, mild fibrosis, moderate fibrosis, and severe fibrosis. Serum and intrahepatic levels of IP-10, IFN-γ, and IL-4 were examined, from which the IFN-γ/IL-4 ratio was calculated. We found that the serum IP-10 levels were positively correlated with the severity of liver fibrosis, whereas the IFN-γ/IL-4 ratio was negatively associated with the progression of hepatic fibrosis. Multivariate logistic regression analysis revealed that the serum IP-10 was an independent predictor for significant fibrosis. For predicting significant fibrosis, the IP-10 cut-off value of 300 ng/mL had a sensitivity of 92.7% and a specificity of 68.6%. When the IP-10 level was combined with the IFN-γ/IL-4 ratio, the specificity and positive predictive value were 93.8% and 94.6%, respectively; thus, the discriminatory ability was much improved. In conclusion, the serum IP-10 level and the IFN-γ/IL-4 ratio have great potential to predict significant fibrosis among CHB patients.

Chronic hepatitis B virus (HBV) infection is one of the major causes of serious liver diseases, including liver cirrhosis and hepatocellular carcinoma (HCC), through a complicated course with fibrosis as a middle essential stage^1^,^2^,^3^. Early detection, diagnosis, and appropriate medical intervention are important to slow down or even stop the rapid progression of HBV-related liver fibrosis into cirrhosis and HCC. Liver biopsy has traditionally been considered as the gold standard for assessment of hepatic fibrosis in chronic hepatitis B (CHB) patients^4^, but it is an invasive procedure with several limitations such as sampling errors and intra- and inter-observer variability. And this technique has recently been challenged by the development of several novel noninvasive tests, relying on quantification of serum markers of liver fibrosis, measurement of liver stiffness by imaging techniques, or the combination of these two approaches. The last decade has witnessed the rapid progress in developing serum markers for the prediction and diagnosis of hepatic fibrosis, such as APRI Score, Fibro Test, FIB-4 index, Hui Score, Zeng Score, etc.^5^,^6^. However, most of the evaluations of serum markers have been performed in patients with chronic hepatitis C virus (HCV) infection, whereas there were only limited data on the serum markers for the early detection and diagnosis of HBV-related fibrosis. A study of 284 of HBV patients and 913 of HCV patients was performed to evaluate diagnostic performance of FibroTest, Firbrometre, Hepacore, and APRI, the range of the area under the receiver operator characteristic curve (AUROC) values in predicting significant liver fibrosis were from 0.72 to 0.78^7^. In the two noninvasive models, Hui Score^8^ and Zeng Score^9^, developed for prediction of significant fibrosis in CHB patients, the mean values of the AUROC in diagnosis of significant liver fibrosis were 0.79 and 0.77, respectively. Obviously, the existing noninvasive models of the serum markers showed lower diagnostic performance for prediction of significant liver fibrosis in HBV patients. Thus, novel noninvasive models with higher ability to predict significant liver fibrosis and to determine stage of liver fibrosis are needed to improve care for hepatitis patients, particularly those with HBV infection.

Interferon gamma-inducible protein-10 (IP-10), also known as C-X-C motif ligand 10 (CXCL10), is an interferon (IFN)-γ/α and tumor necrosis factor alpha (TNF-α)-inducible chemokine that is highly expressed by a variety of cells, including hepatocytes, activated T lymphocytes, natural killer cells, and monocytes. IP-10 as a family member of non-ELR α-chemokines that binds to (C-X-C motif) receptor 3 (CXCR3) and participates in the IFN-mediated innate and specific immnune responses via promoting T helper (Th) 1 effector cells in response to IFN, plays a critical role in inflammation^10^,^11^,^12^,^13^,^14^, and is implicated in the development and progression of hepatic fibrosis. It has been reported that serum and intrahepatic IP-10 levels are increased in HCV-replicating cells and patients with HCV infection^15^,^16^,^17^. Furthermore, associations between serum IP-10 and HCV spontaneous clearance have shown the value of serum IP-10 for the early diagnosis of hepatic fibrosis and treatment outcomes with IFN-based therapy in patients with chronic hepatitis C (CHC)^16^,^17^,^18^,^19^,^20^,^21^. Interestingly, the N-terminal truncated, short form of IP-10 (3–77aa), resulting from the post-translational modification by dipeptidyl peptidase-4 (DPP4), did not show any correlation with the outcome in HCV patients treated with Sofosbuvir/Ribavirin, an IFN-free therapy^22^. In contrast to the extensive studies on IP-10 in HCV infection, less is known about the association between IP-10 and chronic HBV infection. Previous studies, including ours, have demonstrated a significant relationship between IP-10 and HBV infection^23^,^24^. Indeed, as we have shown previously, IP-10 is an independent predictor of HBV e antigen (HBeAg) clearance and is significantly associated with liver inflammation and response to Peg-IFN-α therapy in CHB patients^23^. Besides IP-10, IFN-γ has been identified to inhibit liver fibrosis via inhibiting proliferation and activation of hepatic stellate cells (HSCs) and collagen synthesis. In addition, interleukin (IL)-4, which is closely related to the injury-repair process and accumulation of extracellular matrix proteins, plays a critical role in accelerating liver fibrosis by inducing collagen production via an IL-4Rα-STAT6-dependent mechanism. Thus, the imbalance of IFN-γ/IL-4 might be involved in the immune-pathogenesis in CHB fibrosis. In the present study, we built upon our previous findings to determine the differential expression of IP-10 and the IFN-γ/IL-4 ratio in a total of 180 CHB patients in four groups: no fibrosis (F0), mild or minimal fibrosis (F1–2), moderate fibrosis (F3–4), and severe fibrosis (F5–6), with the last two groups defined as having significant fibrosis according to the Ishak fibrosis scale of 0–6^25^. The aim of this study was to evaluate the predictive value of the IP-10 level alone or in combination with the IFN-γ/IL-4 ratio for liver fibrosis progression in patients with chronic HBV infection.

## Results

### Patient characteristics

A total of 180 CHB patients were enrolled in the period from 2012 to 2014 in this study. The 180 patients were between 12 and 60 years of age, with a median age of 35 ± 12 years old, including 95 (53%) males and 85 (47%) females, who were well distributed among the following groups: F0, without fibrosis; F1–2, with minimal or mild fibrosis; F3–4, with moderate fibrosis; and F5–6, with severe fibrosis. The patients were not co-infected with HAV, HCV, HEV, EBV, CMV, or HIV, nor previously diagnosed with DILI, ALD, or NAFLD, as described in the Materials and Methods section. There were no significant differences of white blood cell count or serum HBV DNA load (Log10 cp/mL) determined at baseline among the groups. The biochemical and virological characteristics were also compared among the CHB patients in the F0, F1–2, F3–4, and F5–6 groups (Table 1). Statistical analysis indicated that ALT, AST, TBil, PT, and INR were significantly higher in all fibrotic groups compared with the controls (F0), with the highest values observed in the F5–6 group; whereas PLT, PTA, and HBsAg declined gradually, with the lowest values found in the F5–6 group. However, there was no significant difference of albumin or TBil between the F0 and F1–2 groups.

### Association of the serum IP-10 level and the IFN-γ/IL-4 ratio with hepatic fibrosis in CHB patients

**Figure 1:**
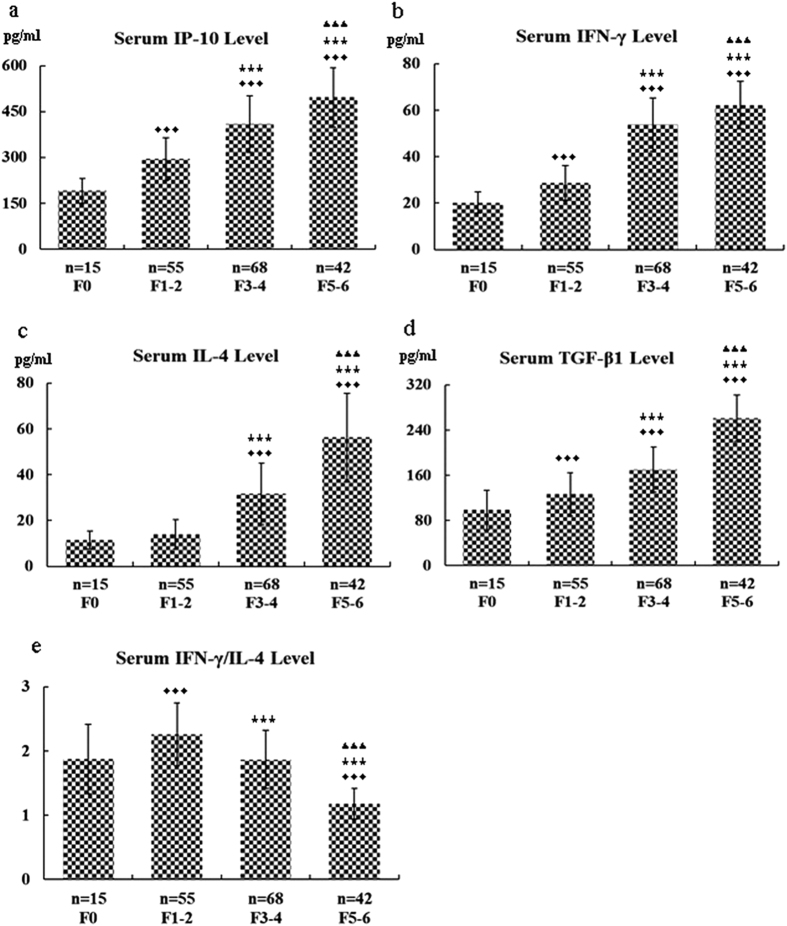
Serum levels of IP-10, IFN-γ, IL-4, and TGF-β1 as well as the IFN-γ/IL-4 ratio in chronic hepatitis B patients with or without fibrosis. The levels of serum IP-10, IFN-γ, IL-4, and TGF-β1 in CHB patients with or without liver fibrosis were determined by ELISA, and the IFN-γ/IL-4 ratio was calculated. ^♦♦♦^Differs from controls (the F0 group), P < 0.05; ^★★★^differs from mild or minimal fibrosis (the F1–2 group), P < 0.05; ^▴▴▴^differs from moderate fibrosis (the F3–4 group), P < 0.05. (**a**) IP-10; (**b**) IFN-γ; (**c**) IL-4; (**d**) TGF-β1; (**e**) the IFN-γ/IL-4 ratio.

We examined the levels of serum IP-10, IFN-γ, IL-4, and transforming growth factor (TGF)-β1 in CHB patients with or without liver fibrosis by ELISA. As shown in Fig. 1, elevated levels of IP-10, IFN-γ, IL-4, and TGF-β1 were detected in the CHB patients with liver fibrosis (F1–2, F3–4, and F5–6), compared to the controls (F0), and there was a trend toward greater levels of IP-10 with an increasing degree of liver fibrosis. The two other fibrosis-associated cytokines (IFN-γ and IL-4) were also enhanced. The differences for serum IP-10, IFN-γ, IL-4, and TGF-β1 between groups and healthy controls were statistically significant (P < 0.05), except there was no significant difference of IL-4 between the F0 group and the F1–2 group (P > 0.05). In contrast to the significant increase in the serum IFN-γ and IL-4 levels in the CHB patients with fibrosis compared to the controls, the ratio of IFN-γ/IL-4 was significantly lower in the F5–6 group than in the F3–4 and F1–2 groups, respectively (P < 0.05), suggesting a negative correlation with the degree of fibrosis. Moreover, a significant increase in the IP-10 levels and a reduction in the ratio of IFN-γ/IL-4 were observed in the significant fibrosis (F3–6) group compared to the mild fibrosis (F1–2) group (P < 0.05).

**Figure 2:**
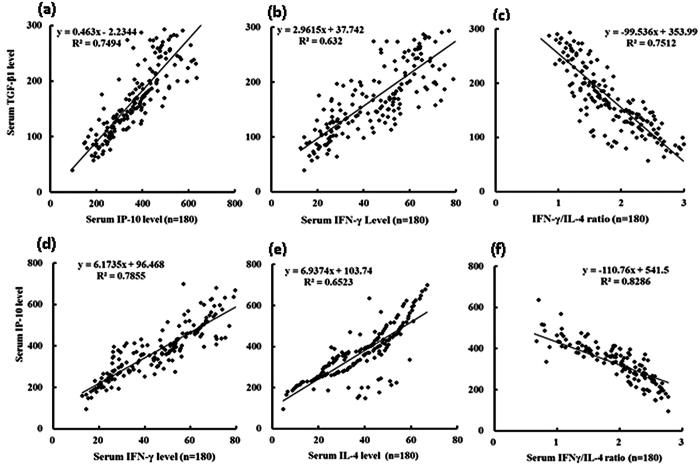
Statistical analysis of the correlation between the serum IP-10 level or the IFN-γ/IL-4 ratio with liver fibrosis among chronic hepatitis B patients. Spearman’s correlation analysis of the association between (**a**) IP-10; (**b**) IFN-γ; (**c**) the IFN-γ/IL-4 ratio and TGF-β1. Spearman’s correlation analysis of the association between serum (**d**) IFN-γ; (**e**) IL-4; (**f** ) the IFN-γ/IL-4 ratio and IP-10.

Spearman’s correlation analysis was performed to analyze the correlation among serum IP-10, the ratio of IFN-γ/IL-4, and TGF-β1 in CHB patients. As shown in Fig. 2, the serum IP-10 and IFN-γ levels showed a positive correlation with the TGF-β1 level (r^2^ = 0.7494 and 0.6320, respectively, P < 0.05), whereas the ratio of IFN-γ/IL-4 and the TGF-β1 level were significantly negatively correlated (r^2^ = 0.7512, P < 0.05). In addition, the serum IP-10 level showed a positive correlation with the IFN-γ and IL-4 levels (r^2^ = 0.7855 and 0.6523, respectively, P < 0.05), whereas the ratio of IFN-γ/IL-4 and the IP-10 level were significantly negatively correlated (r^2^ = 0.8286, P < 0.05).

**Figure 3:**
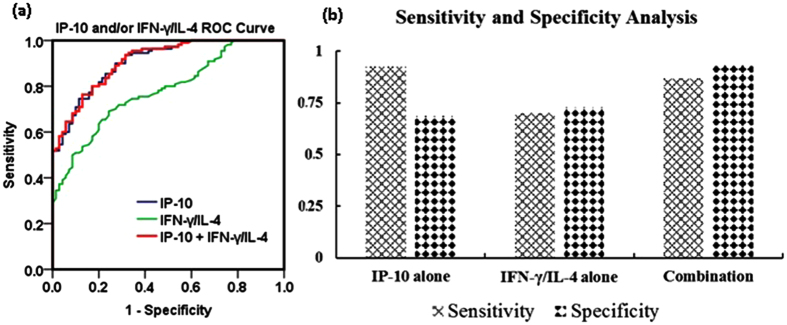
ROC curve analysis for evaluating the sensitivity and specificity of the IP-10 level, IFN-γ/IL-4 ratio, or their combination to predict significant fibrosis among CHB patients. (**a**) ROC curve analysis for serum IP-10 (with the cut-of f value of 300 pg/mL), the serum IFN-γ/IL-4 ratio (with the cut off value of 1.8), and the combination of IP-10 and the IFN-γ/IL-4 ratio; (**b**) Specificity and sensitivity for IP-10, the IFN-γ/IL-4 ratio, and their combination to predict significant liver fibrosis among patients with CHB.

ROC curve analysis was conducted to predict significant liver fibrosis in CHB patients. Furthermore, the area under the ROC curve (AUC) was calculated to evaluate the predictive value of the IP-10 level and the IFN-γ/IL-4 ratio for significant fibrosis in CHB patients. As shown in Fig. 3, ROC curve analysis demonstrated a higher AUC for IP-10 of 0.908 than that for the IFN-γ/IL-4 ratio of 0.780 (P < 0.05). Furthermore, ROC curve analysis identified that a IP-10 level of 300 pg/mL was the most useful cut-off point (with the optimal Youden index) for predictive diagnosis of significant liver fibrosis, with a sensitivity of 92.7%, a specificity of 68.6%, a positive predictive value of 82.3%, and a negative predictive value of 85.7%. The cut-off point for the serum IFN-γ/IL-4 ratio was also identified to be 1.8 (with the optimal Youden index), and this ratio revealed a sensitivity of 70.0%, a specificity of 72.9%, a positive predictive value of 80.2%, and a negative predictive value of 60.7% in predicting significant liver fibrosis. Furthermore, the IP-10 level in combination with the IFN-γ/IL-4 ratio had a sensitivity of 86.9%, a specificity of 93.8%, a positive predictive value of 94.6%, and a negative predictive value of 84.9% for the prediction of significant liver fibrosis in CHB patients. In particular, these data suggest that the IP-10 level combined with the IFN-γ/IL-4 ratio had a considerably greater specificity and predictive value than the IP-10 level or the IFN-γ/IL-4 ratio alone.

Independent factors associated with hepatic fibrosis were also examined in CHB patients with liver fibrosis using binary logistic regression analysis. Initial single index comparison showed that the differences of Alb, ALT, TBil, PT, PTA, PLT, HBV DNA, IFN-γ, IL-4, IP-10, TGF-β, and α-SMA in the mild fibrosis (F1–2) group versus the significant fibrosis (F3–6) group were statistically significant (P < 0.05). However, we subsequently excluded PTA, TGF-β, and α-SMA from the candidate independent factors because PTA was calculated from PT, and TGF-β and α-SMA are well-established factors associated with liver fibrosis. In addition, the ratio of IFN-γ/IL-4 rather than each alone was used because the ratio may reflect the balance of the Th1/Th2 immune system. As shown in Table 2, significant differences in the levels of IP-10, PT, and PLT in CHB patients with mild (F1–2) vs. significant (F3–6) fibrosis were observed by logistic stepwise regression analysis, in which the levels of Alb, ALT, TBil, PT, PLT, HBV DNA, and IP-10 as well as the IFN-γ/IL-4 ratio were involved. Therefore, these four important variables were revealed as independent factors for predicting significant liver fibrosis in CHB patients. The logistic regression equation is given as follows: Logit *P* = −1.125 + 3.365 IP-10 + 1.609 IFN-γ/IL-4 ratio −2.205 PT −2.694 PLT.

### Intrahepatic mRNA and protein levels of IP-10, IFN-γ, and IL-4 in CHB patients

**Figure 4:**
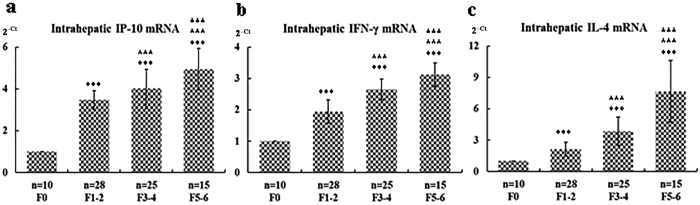
Intrahepatic mRNA levels of IP-10, IFN-γ, and IL-4 in chronic hepatitis B patients with or without fibrosis. Real-time qRT-PCR was conducted to quantify the mRNA levels of intrahepatic IP-10, IFN-γ, and IL-4 in the CHB patients without or with fibrosis as described in the Materials and Methods section. The relative mRNA levels of intrahepatic IP-10, IFN-γ, and IL-4 were calculated by comparative Ct analysis after normalization for the quantity of GAPDH in the same samples and were represented as 2^-△△Ct^ values for controls (the F0 group), which were set equal 1. ^♦♦♦^Differs from controls (the F0 group), P < 0.05; ^★★★^differs from mild or minimal fibrosis (the F1–2 group), P < 0.05; ^▴▴▴^differs from moderate fibrosis (the F3–4 group), P < 0.05. **(a)** IP-10; **(b)** IFN-γ; **(c)** IL-4.

As shown in Fig. 4, the intrahepatic mRNA levels of IP-10, IFN-γ, and IL-4 were analyzed by real-time qRT-PCR in a total of 78 patients who underwent a percutaneous liver biopsy and had enough remaining hepatic tissue for RNA extraction. The intrahepatic IP-10 mRNA expression was significantly upregulated in the CHB patients with fibrosis (F1–2, F3–4, and F5–6) versus the controls (F0) (P < 0.05). The upregulation of IP-10 mRNA was accompanied by a significant increase in IFN-γ and IL-4 mRNA expression (Fig. 4B,C). Moreover, the mRNA levels of IP-10, IFN-γ, and IL-4 increased the most in the F5–6 group, and there was a trend toward higher mRNA levels with the degree of liver fibrosis.

**Figure 5:**
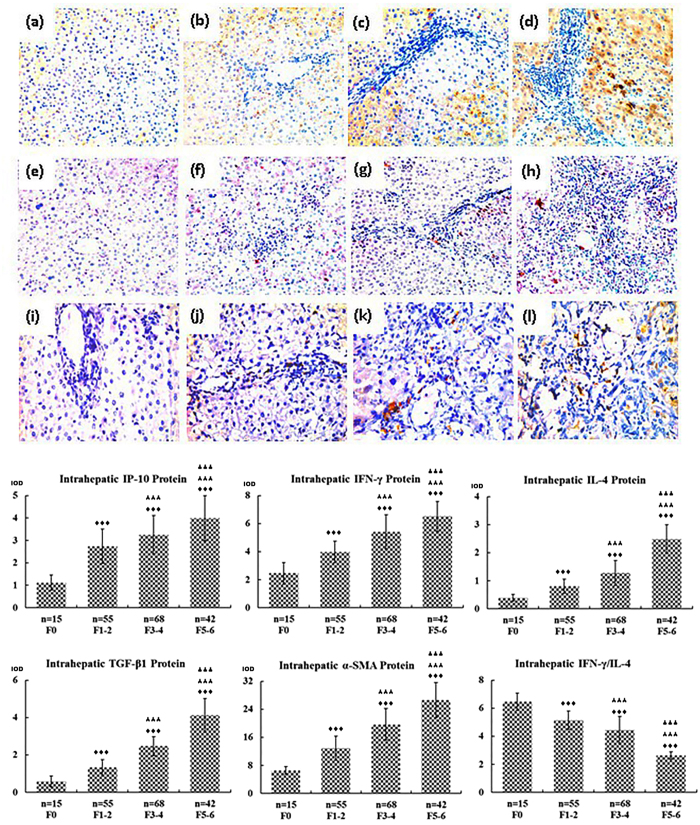
Intrahepatic protein expression of IP-10, IFN-γ, IL-4, TGF-β1, and α-SMA as well as the IFN-γ/IL-4 ratio in chronic hepatitis B patients with or without fibrosis. The protein expression of intrahepatic (a, b, c, and d) IP-10, (e, f, g, and h) IFN-γ, and (i, g, k, and l) IL-4. In addition, the protein levels of intrahepatic IP-10, IFN-γ, IL-4, TGF-β1, and α-SMA were quantified based on the value of integrated optical density (IOD) and represented as histograms, from which the IFN-γ/IL-4 ratio was calculated. ^♦♦♦^Differs from controls (the F0 group), P < 0.05; ^★★★^differs from mild or minimal fibrosis (the F1–2 group), P < 0.05; ^▴▴▴^differs from moderate fibrosis (the F3–4 group), P < 0.05.

Next, we performed experiments using quantitative immunohistochemistry to examine hepatic IP-10, IFN-γ, and IL-4 protein expression in CHB patients with or without liver fibrosis. As shown in Fig. 5, hepatic IP-10 protein was mainly expressed by hepatocytes, particularly in areas of inflammation and necrosis, and around the portal triads. IP-10 protein was also detected in the liver-infiltrating lymphocytes. Compared to the controls (F0 group), IP-10, IFN-γ, and IL-4 protein expression increased with advancing stages of liver fibrosis, and the highest levels of these proteins were found in the F5–6 group, which was consistent with the mRNA analysis results. The difference in mRNA and protein levels between any two liver fibrosis groups of CHB patients was statistically significant (P < 0.05). Whereas the IFN-γ/IL-4 ratio gradually declined with increasing stages of liver fibrosis in the CHB patients, although the mRNA and protein levels of IFN-γ and IL-4 each alone were increased. The difference in the IFN-γ/IL-4 ratio between any two liver fibrosis groups of CHB patients was statistically significant (P < 0.05) (Fig. 5).

## Discussion

HBV infection is one of the major causes of liver fibrosis. The identification of factors associated with HBV-associated hepatic fibrosis has potentially important clinical and pathophysiological significance. This study of a large sample of CHB patients with or without various degrees of hepatic fibrosis had the following major novel findings: (1) The IP-10 level was associated with the degree of liver fibrosis in the CHB patients as demonstrated by several lines of evidence: (a) The serum IP-10 levels were significantly elevated in the CHB patients with moderate to severe fibrosis compared to the CHB patients without fibrosis (Figs 1a and 2a); (b) the intrahepatic IP-10 mRNA and protein levels were significantly upregulated in the CHB patients with fibrosis versus those without hepatic fibrosis (Figs 4 and 5). (2) The IFN-γ/IL-4 ratio was significantly decreased in the CHB patients with fibrosis compared to those without hepatic fibrosis (Figs 1e and 4). (3) The discriminatory ability was significantly improved when the IP-10 level was combined with the IFN-γ/IL-4 ratio Fig. 3. Our results suggest that the serum IP-10 level and the IFN-γ/IL-4 ratio are able to predict significant fibrosis among CHB patients (Fig. 2 and 3).

IP-10 is an IFN-stimulated gene that is induced by IFN-γ/α and TNF-α. In fact, the promoter/enhancer region of the IP-10 gene harbors an IFN-stimulated response element. Multiple studies have shown that IP-10 plays an important role in the development and progression of liver disease, in which IP-10 binds to chemokine CXCR3 and recruits activated T lymphocytes to the liver parenchyma^26^,^27^,^28^. To date, investigations of IP-10 and its correlations with viral clearance and liver fibrosis have been mainly focused on HCV-infected patients^16^,^17^,^18^,^19^,^20^,^21^. High IP-10 levels at acute HCV detection are associated with failure to clear HCV spontaneously, and patients with low levels of IP-10 should defer therapy for potential spontaneous clearance^18^. The IP-10 levels are higher in patients with chronic HCV infection than in healthy controls, and they are positively associated with the severity of liver fibrosis in CHC patients. Until now, limited studies on IP-10 have been performed in HBV-infected patients^23^,^24^,^29^. Previously, we have reported that the serum IP-10 level is an independent predictor of HBeAg clearance and is significantly associated with liver inflammation and the response to Peg-IFN-α therapy in HBV-infected patients^23^. In addition, we have found in our previous work that IP-10 expression distinctly varies at different clinical stages of HBV infection and that higher serum IP-10 expression pretreatment and dynamic downregulation may be associated with an increased probability of HBeAg clearance and HBsAg decline in CHB patients during Peg-IFN-α therapy, suggesting an association of increased levels of serum IP-10 with heavier viral load of HBV^23^. Moreover, it has been demonstrated that a higher expression of CXCR3 in peripheral blood and monocytes is positively correlated with a greater degree of liver fibrosis^23^,^24^. In the present study, we further investigated the IP-10 levels in patients with chronic HBV infection. Our findings in this present study are in agreement with reports that serum fibrosis markers have similar accuracies in predicting advanced fibrosis or cirrhosis in patients with chronic HBV infection as those with chronic HCV infection. In addition, we showed that IP-10 alone had a sensitivity of 92.7% and that IP-10 in combination with the IFN-γ/IL-4 ratio had a specificity of 93.8%; these values are slightly better than those of other noninvasive serum markers for predicting significant fibrosis in CHB patients^30^.

Th cells are involved in immune regulation of CHB patients, which are classified into two different subsets: Th1 and Th2, based on cytokine production. The Th1 and Th2 cytokines not only promote proliferation but also inhibit the differentiation and function of each other so that they maintain immune homeostasis together. In our earlier research, we found that CHB patients who had achieved complete-response after lamivudine therapy showed preferential production of Th1 cells, while nonresponders mainly had a Th2 immune response. However, the role of the Th1/Th2 balance in the development and progression of hepatic fibrogenesis remains obscure. IFN-γ, a cytokine secreted mainly by Th1 cells, has been shown to inhibit liver fibrosis via inhibiting the proliferation and activation of HSCs and collagen synthesis, as well as promoting the activity of natural killer cells that destroy activated HSCs. In contrast, IL-4, which is closely related to the injury-repair process and the accumulation of extracellular matrix proteins, plays a critical role in accelerating liver fibrosis by inducing collagen production via an IL-4Rα-STAT6-depentent mechanism. In this paper, our findings indicate that CHB patients have an imbalanced IFN-γ/IL-4 ratio and that Th1/Th2 cytokines drift into a Th2 lymphocyte subcluster with the degree of liver fibrosis, suggesting that the imbalance of IFN-γ/IL-4 may be involved in the immunopathogenesis of CHB fibrosis. As shown in Fig. 1e, although the negative correlation of IFN-γ/IL-4 ratio with the degree of liver fibrosis in fibrotic CHB patients with score of 1–6, the patients with mild fibrosis (F1–2) showed a significantly higher value of the serum IFN-γ/IL-4 ratio compared to controls with no fibrosis (F0), suggesting there was a positive correlation with fibrosis severity only considering these two groups. These results looked conflicting, that may be attributed to an unparallel increase in FN-γ and IL-4 in different stages of liver fibrosis. IFN-γ, which has been found to play roles in both liver fibrosis and inflammatory reaction, was elevated more than IL-4 in the early stages of liver fibrosis. However, in the late stage of liver fibrosis or liver cirrhosis, IFN-γ increased less than IL-4, which led to the lower ratio of IFN-γ/IL-4 in the F5–6 group versus the F1–2 and F3–4 groups. We also calculated the ratio of IL-4/IFN-γ, and examined the correlation between the ratio of IL-4/IFN-γ with the degree of liver fibrosis, and the resulting data supported the current conclusion as made using the IFN-γ/IL-4 value.

While the present study has supplied much useful information about the predictive value of IP-10 and the IFN-γ/IL-4 ratio in the liver fibrosis of CHB patients, it has several limitations that must be acknowledged. Some other parameters such as TGF-β, platelet also showed correlation with fibrosis severity, we have not compared the specificity, sensitivity of IP-10 or IFN-γ/IL-4 with that of TGF-β. Although an association between IP-10 expression and significant fibrosis among CHB patients was identified, the mechanisms underlying this relationship remain unclear. Based on numerous recent investigations of IP-10 in HCV-infected patients^15^,^16^,^17^,^31^, the expression of IP-10 is specifically increased in HCV-replicating cells upon stimulation with conventional toll-like receptor 2 (TLR2) ligands, subsequently inducing CD44 expression. CD44 is a broadly distributed type I transmembrane glycoprotein and a receptor for the glycosaminoglycan hyaluronan (HA)^32^,^33^,^34^. In CHC patients, the expression of HA in serum has been shown to increase in accordance with the progression of liver fibrosis, and HA also works as a ligand for TLR2. In addition, IP-10 production upon HA stimulation depends on the expression of TLR2 and CD44, and a direct association between TLR2 and CD44 has been observed, suggesting that the endogenous expression of HA in hepatocytes in CHC patients participates in IP-10 production through an interaction between TLR2 and CD44, though an interaction with TLR2 and HA in HBV infection needs further study. Until now, the exact mechanisms that IP-10 participated in the fibrotic progression during chronic HBV infection are not well understood^35^,^36^. A comparative study of IP-10 deficient mice (IP-10^−/−^ and C57BL/6 mice treated with the neutralizing anti-IP-10 antibody) and wildtype C57BL/6 mice showed that CCl_4_-induced liver fibrosis was less severe in the IP-10 deficient mice with massively increased infiltrating NK cells but lower frequency of activated HSCs^37^. Most recently, a study with IP-10 knockout murine model of diet-induced NASH demonstrated that reduction of liver fibrosis was related to IP-10-dependent decrease in hepatic M1 polarized macrophages accumulation and activation^38^. All the results revealed a significant effect of IP-10 on the liver fibrotic progression, which is likely attributed to the NK cell-mediated inactivation of HSCs, as well as macrophages to M1 subtype polarization. More importantly, the recognized mechanism whereby IP-10 aggravates tissue injury favors the recruitment of Th1 dominant cells by binding to its specific cognate receptor CXCR3. However, different IP-10 forms appeared to exert distinct functions. It has been reported that the long-length IP-10 directed CXCR3^+^ T cell migration which was associated with inflammation, while the short IP-10 was a CXCR3 antagonist, thereby playing a protective role in liver injury^39^. This might explain why some contrasting results were obtained in the study of IP-10 on hepatic injury progression. Recently, Liang *et al*.^40^ have examined the roles for IP-10 in the development and progression of liver fibrosis using HSCs *in vitro*. They found that IP-10 suppresses the expression of fibrosis-associated genes in liver nonparenchymal cells, primary HSCs of mice in schistosomiasis, and TGF-β-activated human hepatic stellate LX-2 cells, suggesting an anti-fibrotic role for IP-10 in liver fibrosis. Our studies have shown the positive correlation of IP-10 and the negative correlation of IFN-γ/IL-4 ratio with the degree of liver fibrosis in fibrotic CHB patients with score of 1–6, for which IP-10 in combination with the IFN-γ/IL-4 ratio significantly improved the discriminatory ability in predicting liver fibrosis. However, further studies are needed to find better and more effective serum marker combinations for the early diagnosis of liver fibrosis among CHB patients.

Although these recent studies have advanced our knowledge and have paved ways to insightful studies in the future, further insightful investigations on the role of IP-10 in HBV-induced hepatic fibrosis would be interesting to unlock the molecular mechanisms that might lead to an explanation for elevated IP-10 levels in CHB patients with a higher degree of hepatic fibrosis. As one of our research projects, we have being working on establishing an IP-10 knock-out mouse model to perform an in-depth investigation of molecular mechanisms underlying the role of IP-10 in the liver fibrosis.

In conclusion, our study suggests the great potential for IP-10 alone or in combination with the IFN-γ/IL-4 ratio as a useful tool to prioritize patients or to predict significant fibrosis among patients suffering from CHB. Thus, our findings add an important serum marker, either alone or in combination with the IFN-γ/IL-4 ratio, to predict significant liver fibrosis induced by HBV infection. Further insightful investigations are underway in our laboratory that propose to identify systematically and to determine interactions that regulate cellular gene expression directly related to HBV-induced liver fibrogenesis in cell cultures, animal models, and human subjects.

## Materials and Methods

### Human subjects

A cohort of 180 CHB patients undergoing a liver biopsy was enrolled in this retrospective study between January 2012 and December 2014 at three hospitals, including The Third Hospital of Heibei Medical University, The Infectious Disease Hospital of Handan City, and The First Hospital of Shijiazhuang City. All CHB patients were diagnosed based on the presence of the hepatitis B surface antigen (HBsAg) for at least 6 months, in accordance with the guidelines for the management of CHB by the Asian Pacific Association for the Study of the Liver^41^. In addition, the patients were tested for baseline biochemical and hematological parameters on presentation and matched the following criteria: a HBV DNA level of more than 1,000 copies/mL, serum alanine aminotransferase (ALT) level of less than 10 times the upper limit of normal (ULN), serum total bilirubin (TBil) concentration of less than 35 μM, and extension of prothrombin time (PT) of less than 3 s prior to recruitment. According to the Ishak fibrosis scale of 0–6, ranging from normal to cirrhosis, the patients were categorized into four groups: F0 (no fibrosis as control, n = 15), F1–2 (mild or minimal fibrosis, n = 55), F3–4 (moderate fibrosis, n = 68), and F5–6 (severe fibrosis, n = 42), in which F3–6 was defined as significant fibrosis^25^,^42^. To minimize the inter-observer viability in the assessment of liver fibrosis, two blinded expert liver pathologists reviewed all the biopsies and scored liver fibrosis in the 180 CHB patients using the Ishak scoring system. In addition, a few patients with insufficient and unqualified liver biopsies were excluded from the present study to reduce the intra-observer viability. Exclusion criteria for patients included coinfections with hepatitis A virus (HAV), HCV, hepatitis E virus (HEV), Epstein-Barr virus (EBV), cytomegalovirus (CMV), or human immunodeficiency virus (HIV); drug-induced liver injury (DILI); alcoholic liver disease (ALD) or nonalcoholic fatty liver disease (NAFLD); autoimmune liver disease; diabetes; hypertension; involvement of HCC; or pregnancy in women. A percutaneous liver biopsy and serum samples were collected from all patients on the same day that the liver biopsy was performed.

The research protocol was approved by the Human Research Ethics Committee of the Third Hospital of Hebei Medical University. All procedures performed in studies involving human participants were in accordance with the ethical standards of the institutional and national research committee and with the 1964 Helsinki declaration and its later amendments or comparable ethical standards. All patients gave written informed consent to participate in this study after the nature of the study had been explained to them, which included a review of each subject’s medical records and samples.

### Reagents and antibodies

Trizol Reagent was purchased from Invitrogen (Carlsbad, CA, USA), and the PrimeScript^TM^ RT Kit was from Fermentas (Burlington, ON, Canada). The GoTaq^®^ RT-Qpcr System was obtained from Promega (Madison, WI, USA). All primers used in the present study were synthesized and purified by Sangon Biotech (Shanghai, China). Rabbit anti-human IP-10 polyclonal antibody was purchased from Abcam (Cambridge, MA, USA). Rabbit anti-human IFN-γ, IL-4, TGF-β1, and α-smooth muscle actin (SMA) polyclonal antibodies were purchased from Boster (Wuhan, Hubei, China). Secondary antibodies and 3, 3′-diaminobenzidine were from ZSGB-Bio (Beijing, China). The Quantikine Human IP-10 (sensitivity, 4.46 pg/mL; limit of quantification (LOQ), 7.8–600 pg/mL) and TGF-β1 (sensitivity, 15.4 pg/mL; LOQ, 31.2–2,000 pg/mL) ELISA kits were from R&D Systems (Minneapolis, MN, USA); the IFN-γ (sensitivity, 4 pg/mL; LOQ, 7.8–500 pg/mL) and IL-4 (sensitivity, 2 pg/mL; LOQ 3.9–250 pg/mL) ELISA kits were from BioLegend, Inc. (San Diego, CA, USA).

### Specimen collection

An echo-guided percutaneous liver biopsy was performed, and two liver tissues (length of specimen, 1.5–2 cm) were obtained from each participant. From each patient, one biopsy sample was fixed in 4% buffered formaldehyde, embedded into paraffin blocks, and sliced for subsequent liver pathology and immunohistochemistry assays; while the remaining liver biopsy samples, which were available in 78 (43%) subjects due to a limited amount of the liver biopsy, were stored in RNAlater for total RNA isolation and then subsequent real-time quantitative reverse transcription polymerase chain reaction (qRT-PCR) assays.

### Biochemical analyses

The white blood cell and platelet (PLT) counts were determined from blood samples using an Automatic Blood Cell Counter Plus. Serum albumin, ALT, aspartate aminotransferase (AST), and TBil levels were measured with an Automatic Biochemical Analyzer. PT was examined using an Automatic Coagulometer from which the prothrombinase activity and the International normalized ratio (INR) were calculated. Automatic Architect assays were used to quantify the levels of HBsAg and HBeAg on an I2000 assay platform (Abbott Diagnostics, USA), with a lower limit of detection for HBsAg of 0.05 IU/mL and for HBeAg of 1.0 S/Co. Determination of the serum HBV DNA loads were carried out by real-time PCR on an ABI 7500 sequence detector (Applied Biosystems, USA). Serum IP-10, IFN-γ, IL-4, and TGF-β1 levels were determined by ELISA, according to the manufacturer’s instructions.

### Real-time qRT-PCR of IP-10, IFN-γ, and IL-4 mRNA levels

Total RNA was extracted from the entire RNAlater-stored liver tissue using Trizol Reagent. cDNA synthesis by reverse transcription was performed using a PrimeScriptTM RT Kit, according to the manufacturer’s instructions. The primers used in this study were as follows: IP-10-specific sense primer, 5′-GCC TCT CCC ATC ACT TCC CTA C-3′ (22 bp), anti-sense primer, 5′-GAA GCA GGG TCA GAA CAT CCA C-3′ (22 bp); IFN-γ-specific sense primer, 5′-CTC ATG TAA GCC CCC AGA AA-3′ (20 bp), anti-sense primer, 5′-GCC CAG TTC CTG CAG AGT AG-3′ (20 bp); IL-4-specific sense primer, 5′-GCC TTC AGC ACA TCT TCA CA-3′ (20 bp), anti-sense primer, 5′-ATC ATC GCT TCT CTG CAC CT-3′ (20 bp); GAPDH-specific sense primer, 5′-ACC ACA GTC CAT GCC ATC ACT-3′ (21 bp), anti-sense primer, 5′-TCC ACC ACC CTG TTG CTG TA-3′ (20 bp). Real-time quantitative RT-PCR was performed using a GoTaq^®^ RT-qPCR System from Promega, according to the manufacturer’s instructions. Samples without template and reverse transcriptase were included and served as negative controls, which expectedly produced negative signals (Ct values > 35). Relative mRNA levels of IP-10, IFN-γ, and IL-4 were calculated by comparative threshold cycle (Ct) analysis after normalization for the quantity of GAPDH in the same samples and were represented as 2^−△△Ct^ values, which were transformed from the initial Ct values and calculated as follows: ΔCt (F1–2, F3–4, or F5–6 group) = Ct test gene (IP-10, IFN-γ, or IL-4) - Ct reference gene (GAPDH); ΔCt (Control F0 group) = Ct test gene (IP-10, IFN-γ, or IL-4) - Ct reference gene (GAPDH); ΔΔCt = ΔCt (F1–2, F3–4, or F5–6)−ΔCt (F0).

### Immunohistochemistry

Immunohistochemical reactions using the Power Vision^TM^ Two**-**Step Detection System were performed to examine the protein expression of IP-10, IFN-γ, IL-4, TGF-β1, and α-SMA in liver tissue sections, according to a standard protocol in our laboratory, as described previously^23^. The immunoreactivity was quantified by outlining the whole liver sections using Image Pro Plus software (Media Cybernetics, Silver Spring, MD, USA) to determine the values of integrated optical density (IOD).

### Statistical analysis

The results of each group were given as mean ± standard deviation. All statistical analyses were performed with IBM SPSS Statistics version 21.0 from SPSS Inc. (Chicago, IL, USA). HBV DNA loads were logarithmically transformed. Categorical variables were analyzed by Pearson’s chi-squared test. Continuous variables were initially verified by normal distribution (Kolmogroc-Smirnov test) and the homoscedasticity (Levene test) test. Statistical significance was subsequently determined by the nonparametric Kruskal-Wallis H test, followed by the Mann-Whitney U test for pairwise comparisons. Furthermore, spearman linear correlation tests were performed to evaluate the correlation between TGF-β1 with IP-10, IFN-γ, and the IFN-γ/IL-4 ratio. Stepwise binary logistic regression analysis was used to determine whether the identified variables associated with histological features of fibrosis were independent risk factors for significant fibrosis. Receiver operating characteristic (ROC) curve analysis was used to evaluate the diagnostic accuracy of the serum IP-10 level in predicting the extent of fibrosis. A two-sided P value of <0.05 was considered statistically significant.

## Additional Information

**How to cite this article**: Wang, Y. *et al*. Predictive Value of Serum IFN-γ inducible Protein-10 and IFN-γ/IL-4 Ratio for Liver Fibrosis Progression in CHB Patients. *Sci. Rep.* **7**, 40404; doi: 10.1038/srep40404 (2017).

**Publisher's note:** Springer Nature remains neutral with regard to jurisdictional claims in published maps and institutional affiliations.

## Supplementary Material

Supplementary Information

## Figures and Tables

**Table 1:** Characteristics of the 180 chronic hepatitis B patients with or without hepatic fibrosis.

Characteristic	F0	F1–2	F3–4	F5–6
n	15	55	68	42
Mean age, years	31 ± 10	35 ± 10	34 ± 12	38 ± 14
Male/Female, n	7/8	28/27	36/32	24/18
WBC, ×10^9^/L	6.82 ± 1.44	6.26 ± 1.52	6.38 ± 1.67	6.20 ± 2.14
PLT, ×10^9^/L	224 ± 43	159 ± 45^a^	156 ± 40^a^	127 ± 33^abc^
Albumin, g/L	42.61 ± 4.20	40.69 ± 4.81	40.81 ± 4.82	37.61 ± 4.16^abc^
Serum ALT, U/L	49 ± 23	97 ± 38^a^	124 ± 42^ab^	118 ± 55^ab^
Serum AST, U/L	47 ± 18	83 ± 33^a^	81 ± 39^a^	93 ± 38^a^
Serum TBil, μM	15.57 ± 4.29	15.94 ± 5.23	18.52 ± 5.94^b^	25.55 ± 6.15^abc^
PT, s	11.02 ± 0.42	11.87 ± 0.83^a^	12.17 ± 0.90^a^	12.33 ± 1.22^a^
PTA, %	133.10 ± 17.20	114.52 ± 16.24^a^	109.69 ± 15.82^a^	100.83 ± 15.69^abc^
INR	0.82 ± 0.09	0.93 ± 0.12^a^	0.95 ± 0.12^a^	1.05 ± 0.15^abc^
HBV DNA, Log_10_ cp/mL	6.32 ± 0.36	6.11 ± 0.89	5.85 ± 1.02	5.96 ± 0.80
HBeAg, + /−(n)	12/3	35/20	35/33^a^	18/24^ab^
Serum HBsAg, IU/mL	7325 ± 2546	5102 ± 2810^a^	5277 ± 2957^a^	2438 ± 1776^abc^

WBC, white blood cell; PLT, platelets; ALT, alanine aminotransferase; AST, aspartate aminotransferase; TBil, serum total bilirubin; PT, prothrombin time; PTA, prothrombinase activity; INR, international normalized ratio; HBV, hepatitis B virus; HBeAg, hepatitis B e antigen; HBsAg, hepatitis B surface antigen.

^a^Differs from the F0 group, *P* < 0.05; ^**b**^Differs from the F1–2 group, *P* < 0.05; ^**c**^Differs from the F3–4 group, *P* < 0.05.

**Table 2:** Binary logistic-regression analysis of independent predictors of significant liver fibrosis.

Variable	B	Wald	*P* value	Odds Ratio	95% CI for EXP(B)	
					Lower	Upper
PT level (μM) (<12 = 0; ≥12 = 1)	−2.205	8.813	0.003	0.110	0.026	0.473
PLT level (×10^9^/L) (≥100 = 0; <100 = 1)	−2.694	10.920	0.001	0.068	0.014	0.334
Serum IP-10 levels (pg/mL) (<300 = 0; ≥300 = 1)	3.365	18.025	0.000	28.944	6.121	136.859
IFN-γ/IL-4 ratio (<1.8 = 0; ≥1.8 = 1)	1.609	7.358	0.007	4.996	1.562	15.974
Constant	−1.125	7.361	0.007	0.325		

PT, prothrombin time; PLT, platelets; IP-10, IFN-γ inducible protein, 10 kD; IFN-γ, interferon gamma; IL-4, interleukin 4; CI, confidence interval.
