# Supplementary material for: Predictive Value of Serum IFN-γ inducible Protein-10 and IFN-γ/IL-4 Ratio for Liver Fibrosis Progression in CHB Patients
Source: Sci Rep. 2017 Jan 9;7:40404. doi: 10.1038/srep40404 (PMC5220308; doi:10.1038/srep40404)
Supplement: Supplementary Information [file srep40404-s1.pdf]

Predictive Value of Serum IFN-γ inducible Protein-10 and IFN-γ/IL-4 Ratio for Liver Fibrosis Progression in CHB Patients

Yadong Wang[1], Weiyan Yu[1], Chuan Shen[1], Wei Wang[1], Li Zhang[1], Fang Liu[1], Hui Sun[1], Yajuan Zhao[2], Honghao Che[3], Caiyan Zhao[1*]

Suppl. figure legends

Suppl. Figure 1. Intrahepatic IP-10 expression. Representative micrographs of serial liver tissue sections from the four groups of CHB patient samples: F0 (a), F1–2 (b), F3–4 (c), and F5–6 (d). Magnifications: 200×

Suppl. Figure 2. Intrahepatic IFN-γ expression. Representative micrographs of serial liver tissue sections from the four groups of CHB patient samples: F0 (a), F1–2 (b), F3–4 (c), and F5–6 (d). Magnifications: 200×

Suppl. Figure 3. Intrahepatic IL-4 expression. Representative micrographs of serial liver tissue sections from the four groups of CHB patient samples: F0 (a), F1–2 (b), F3–4 (c), and F5–6 (d). Magnifications: 400×

Suppl. Figures

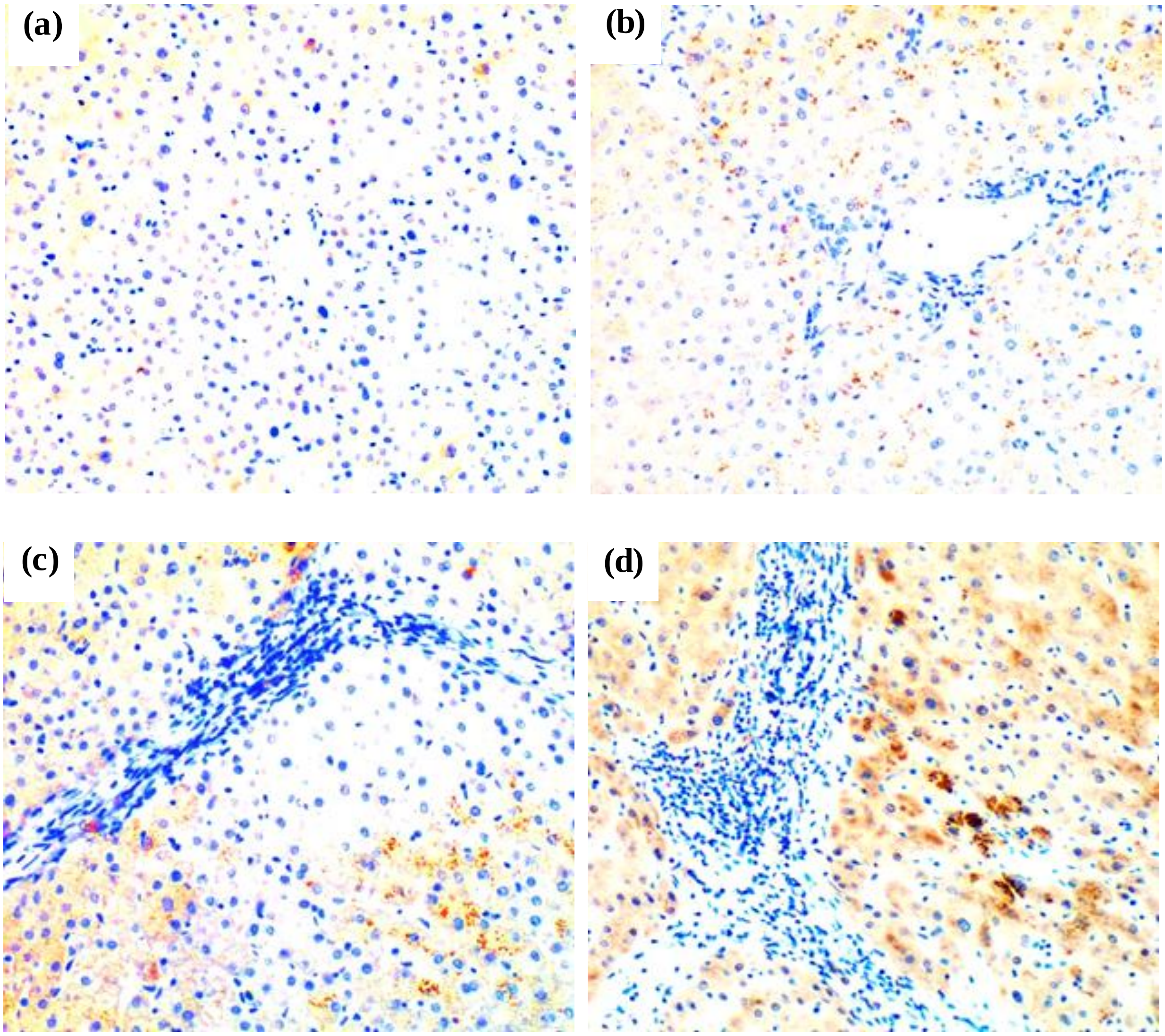

Suppl. Figure 1 Intrahepatic IP-10 expression

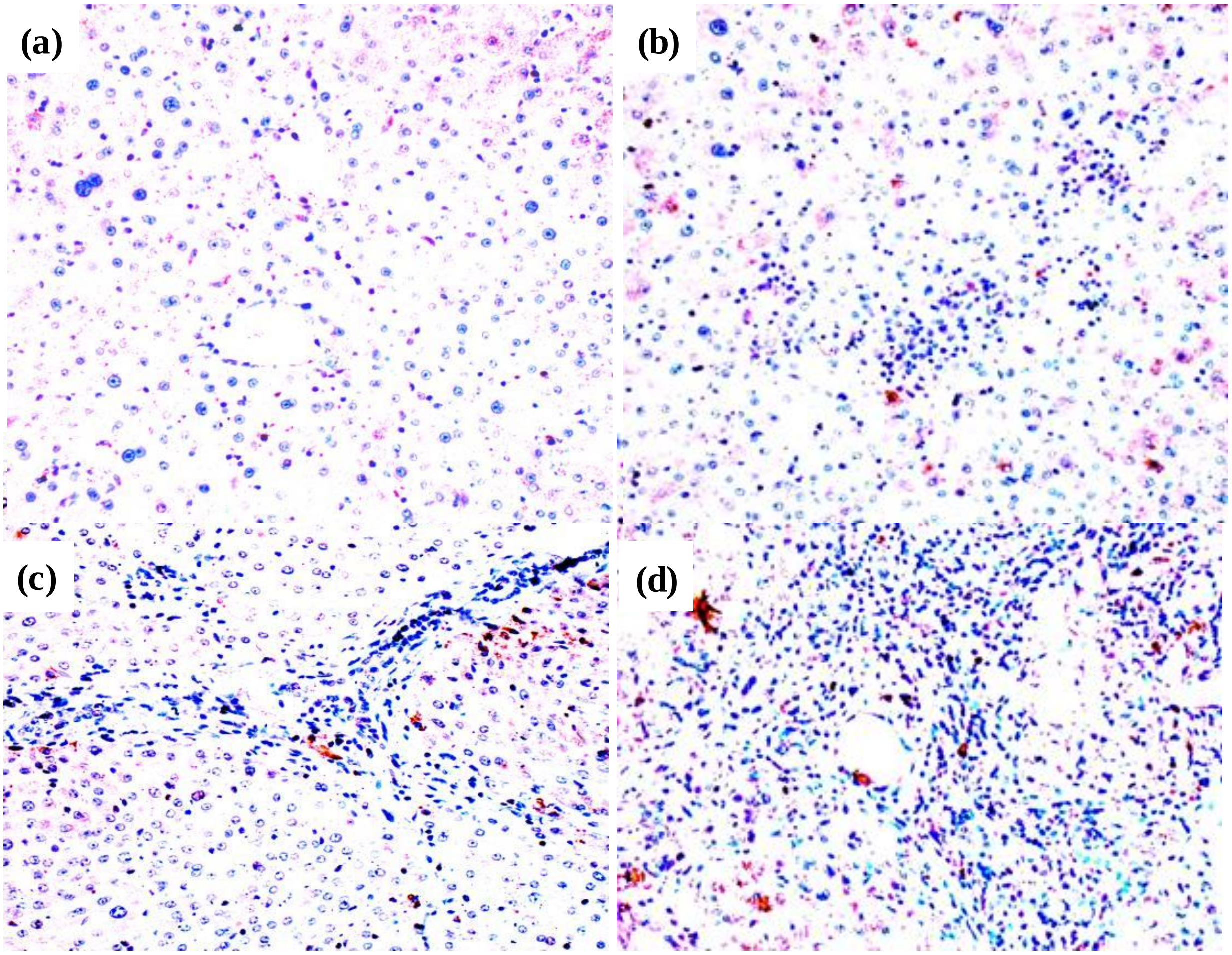

Suppl. Figure 2 Intrahepatic IFN-γ expression

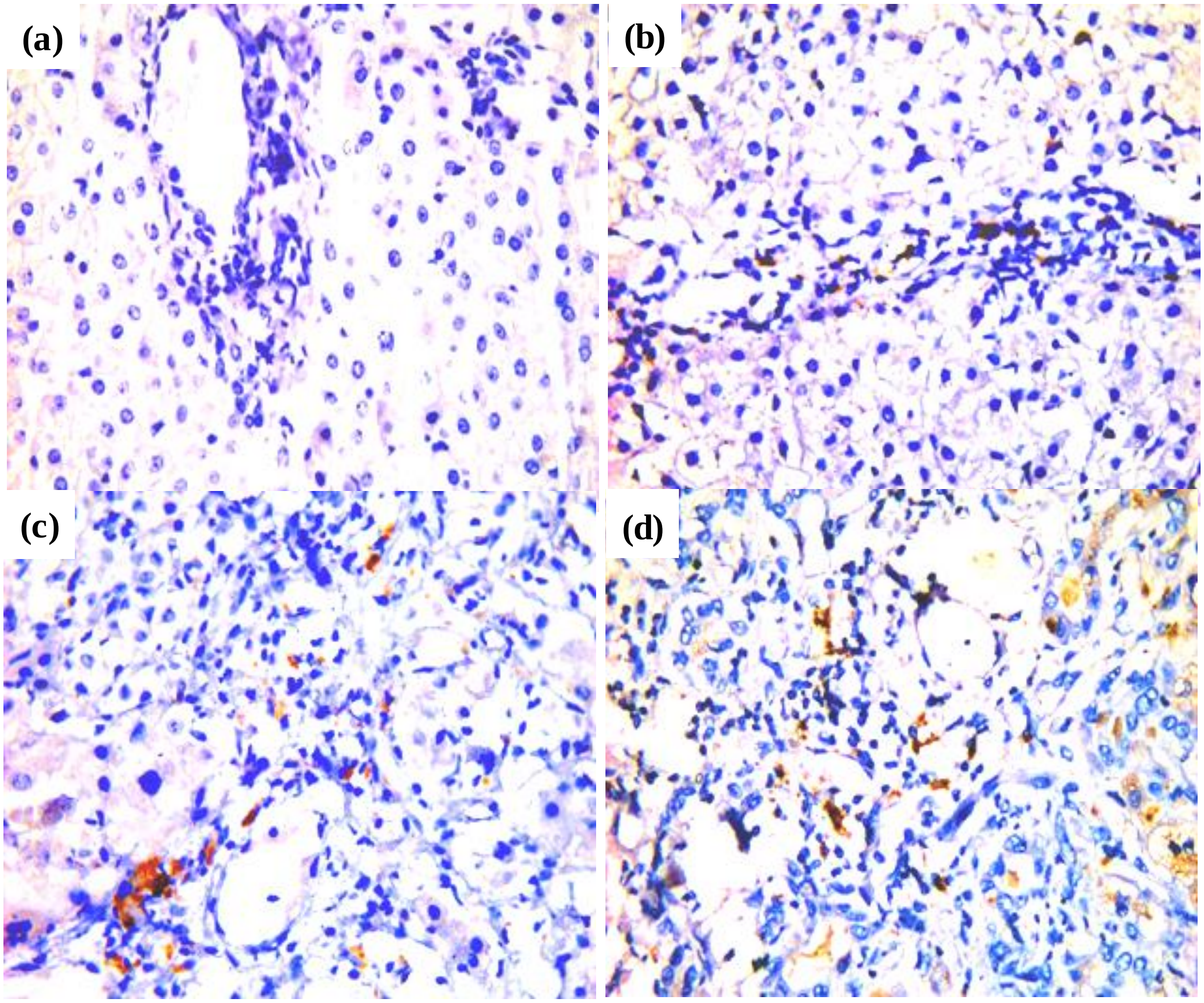

Suppl. Figure 3 Intrahepatic IL-4 expression
